# Supplementary material for: Polymorphism of Carbamazepine Pharmaceutical Cocrystal: Structural Analysis and Solubility Performance
Source: Pharmaceutics. 2023 Jun 15;15(6):1747. doi: 10.3390/pharmaceutics15061747 (PMC10302832; doi:10.3390/pharmaceutics15061747)
Supplement: Supplementary file 1 [file pharmaceutics-15-01747-s001.zip › pharmaceutics-2425598-supplementary.pdf]

# Supporting Information

# Polymorphism of carbamazepine pharmaceutical cocrystal: structural analysis and solubility performance


*Artem O. Surov[1*], Ksenia V. Drozd[1], Anna G. Ramazanova[1], Andrei V. Churakov[2], Anna V. Vologzhanina[3], Elizaveta S. Kulikova[4], German L. Perlovich[1]*

[a]G. A. Krestov Institute of Solution Chemistry RAS, Akademicheskaya str. 1, 153045, Ivanovo, Russia

[b]N. S. Kurnakov Institute of General and Inorganic Chemistry RAS, Leninsky Prosp. 31,119991, Moscow, Russia

[c]A. N. Nesmeyanov Institute of Organoelement Compounds RAS, Vavilova str. 28, 119334 Moscow, Russia

[d]National Research Center Kurchatov Institute, 1 Kurchatova pl., 123098, Moscow, Russia

*Correspondence: aos@isc-ras.ru*

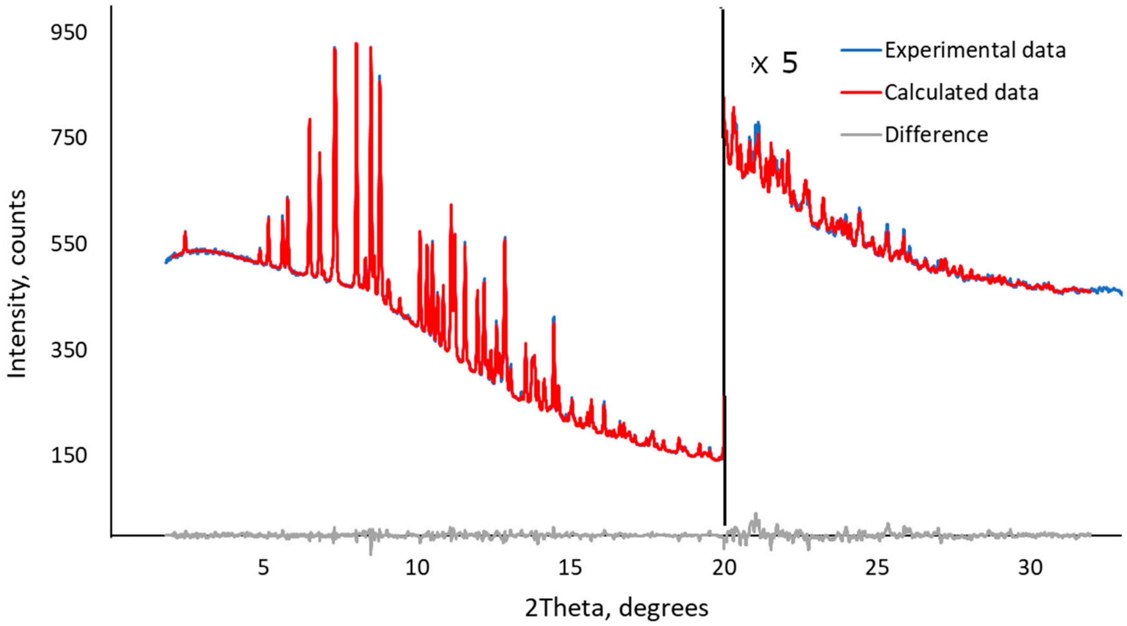


**Figure S1.** Final observed (blue), calculated (red) and difference profiles for the Rietveld refinement of [CBZ+MePRB] (1:1) form II cocrystal

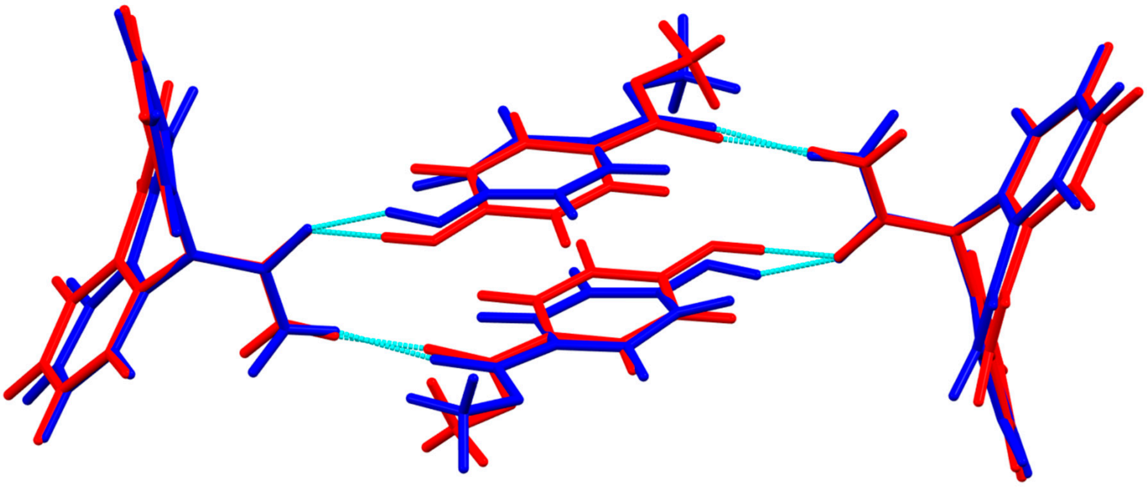

**Figure S2.** A superposition a closed-ring supramolecular tetrameric unit formed by the hydrogen-bonded CBZ and MePRB molecules in the structures of form I (red) and form II (blue).

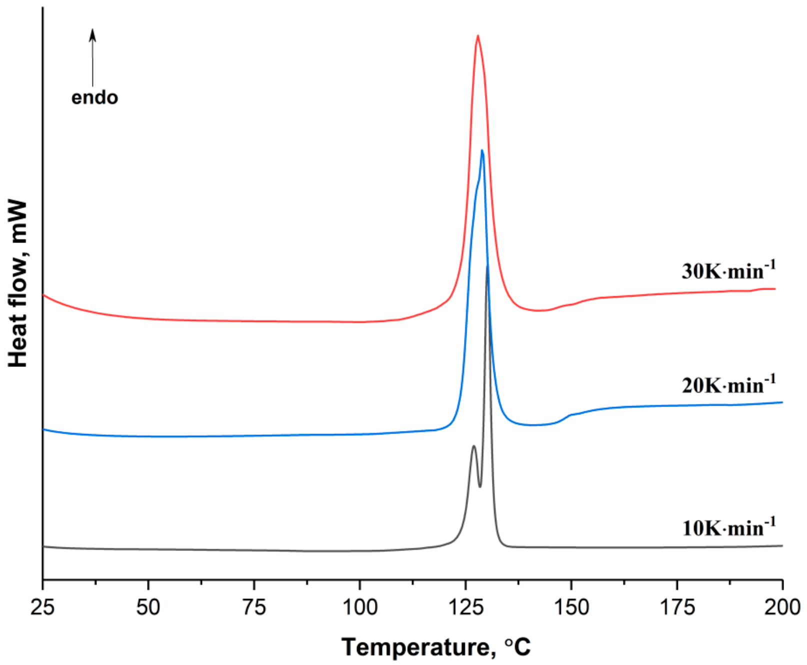


**Figure S3.** DSC traces for [CBZ+MePRB] (1:1) form I recorded at different heating rates

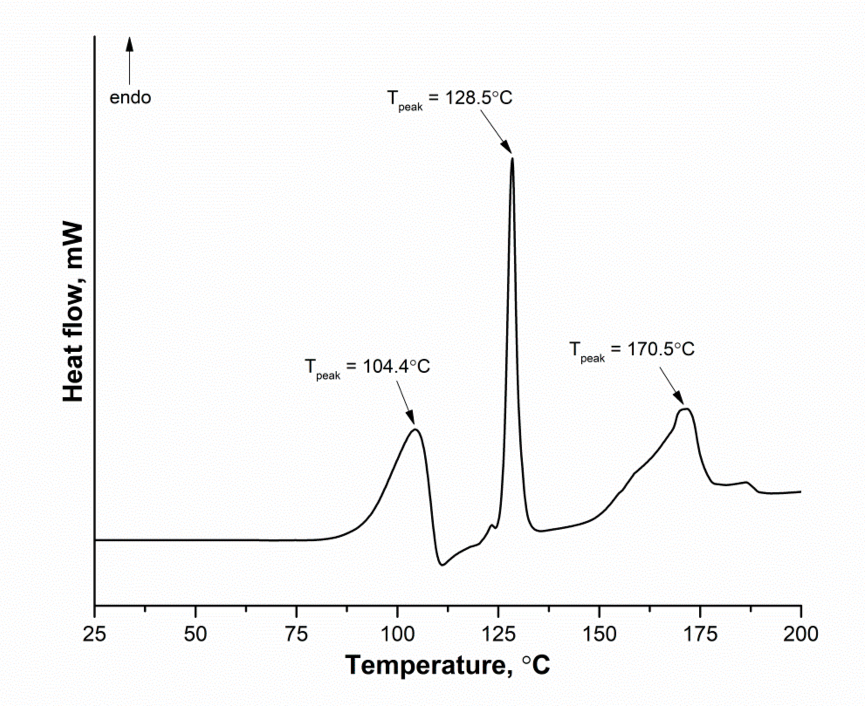


**Figure S4.** DSC trace for the [CBZ+MePRB] (1:0.25) cocrystal.

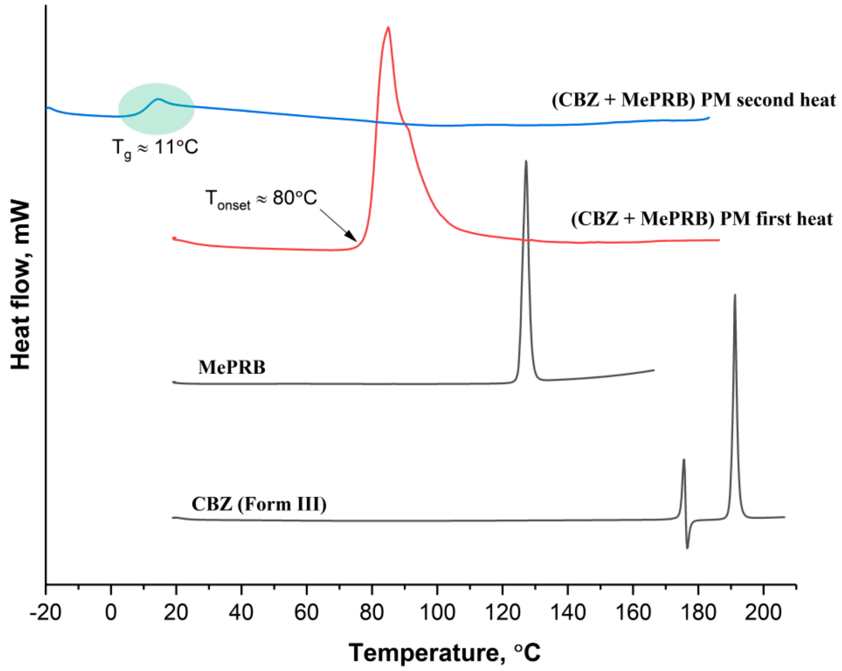


**Figure S5.** Heat-cool-heat cycles for physical mixture (PM) of CBZ and MePRB illustrating eutectic melting and formation of co-amorphous system

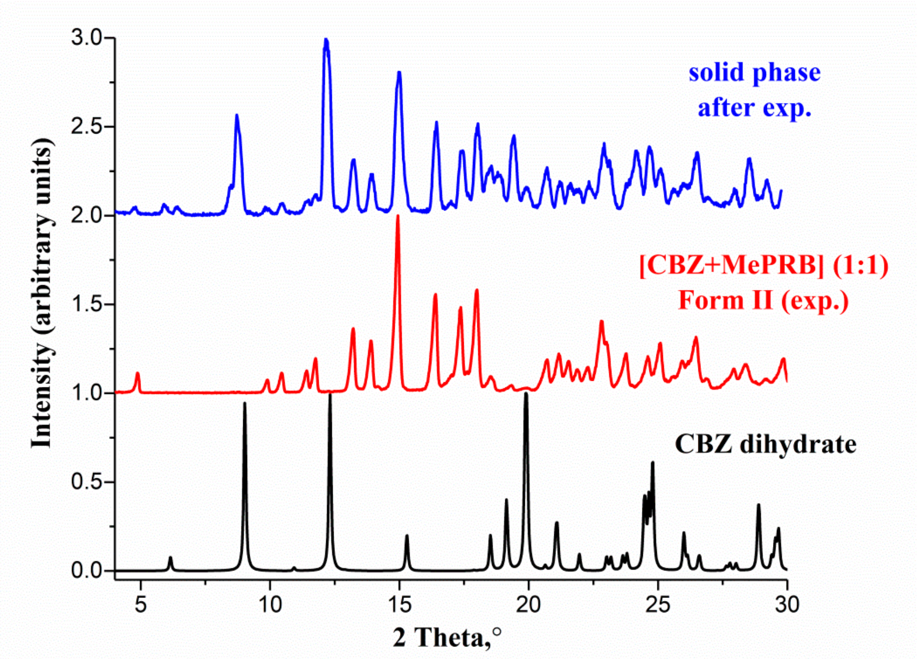


**Figure S6.** Experimental PXRD patterns of CBZ dihydrate, [CBZ+MePRB] (1:1) Form II and solid phase at the eutectic point in a pH 6.5 buffer solution at 37 °C, indicating that both CBZ dihydrate and cocrystal solids are presented in equilibrium with the solution.

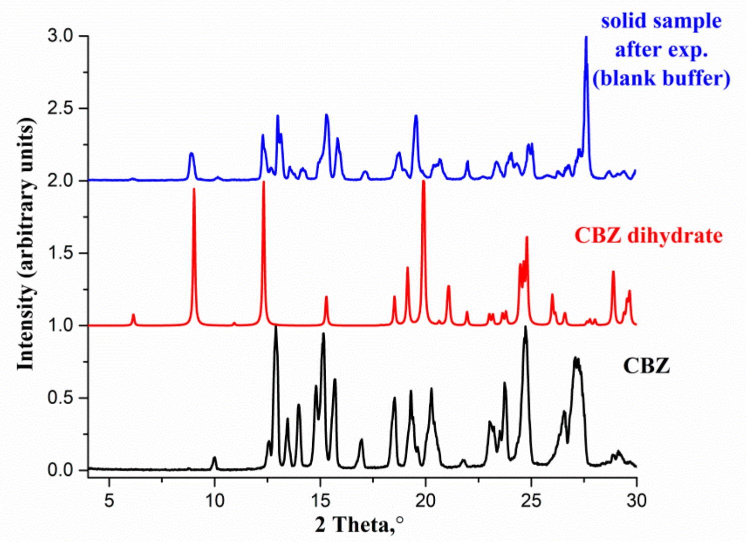


(a)

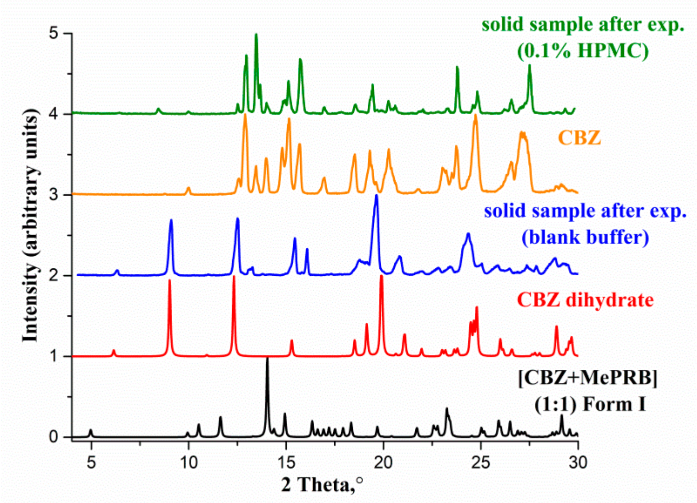


(b)

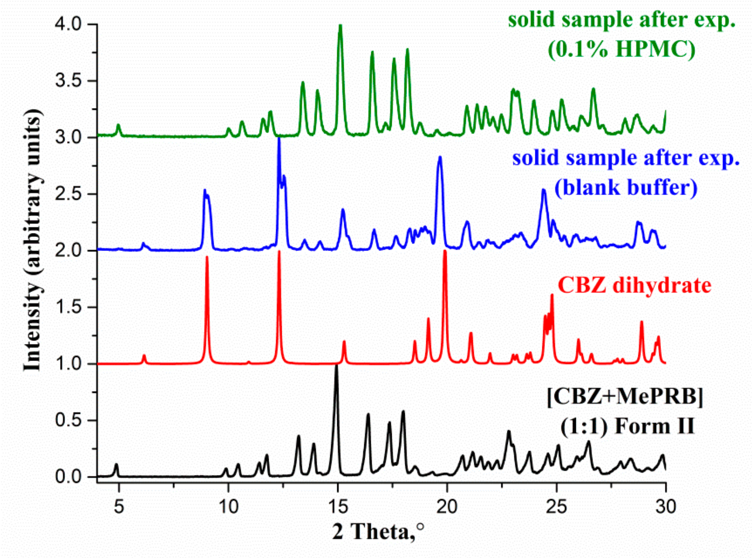


(c)

**Figure S7**. Experimental PXRD patterns of the residual solids after the dissolution experiments for (a) CBZ, (b) [CBZ+MePRB] (1:1) Form I and (c) [CBZ+MePRB] (1:1) Form II in pH 6.5 buffer solution with and without pre-dissolved HPMC polymer (0.1%).

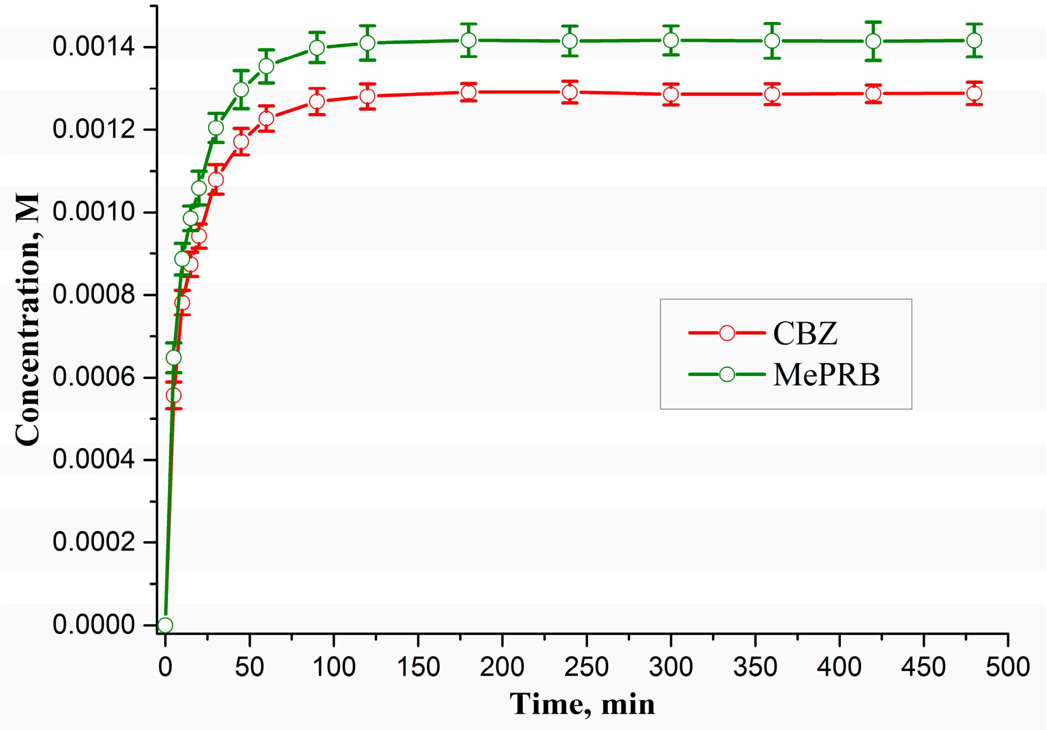


(a)

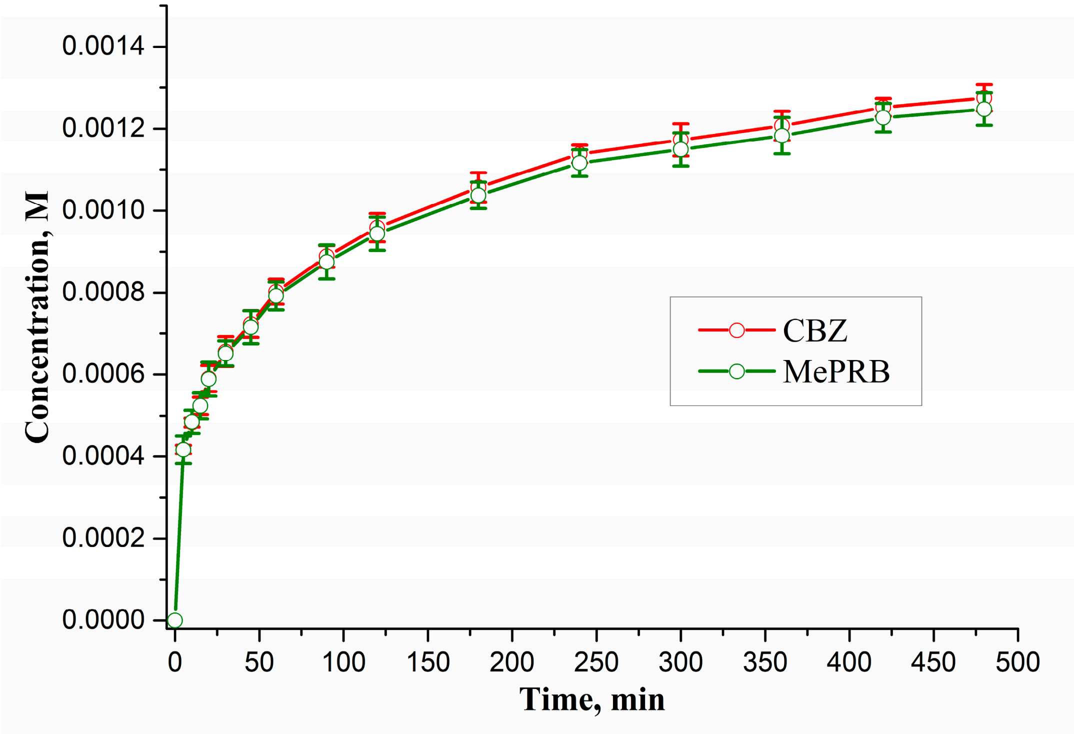


(b)

**Figure S8.** Powder dissolution profiles of CBZ and MePRB obtained as a result of (a) [CBZ+MePRB] (1:1) Form I or (b) [CBZ+MePRB] (1:1) Form II dissolution in 6.5 buffer solution with pre-dissolved HPMC (0.1%).

**Table S1.** Crystallographic data for [CBZ+MePRB] (1:0.25) cocrystal

| Compound reference | [CBZ+MePRB] (1:0.25) |
|---|---|
| CCDC number | 2262553 |
| Chemical formula | $C_{15}H_{12}N_2O\cdot 0.25C_8H_8O_3$ |
| *F*w | 274.30 |
| Crystal system | Monoclinic |
| *a,* Å | 20.4142(14) |
| *b*, Å | 5.1684(4) |
| *c*, Å | 26.3796(19) |
| *α*, ° | 90.00 |
| *β*, ° | 94.810(2) |
| *γ*, ° | 90.00 |
| Unit cell volume, Å$^3$ | 2773.5(3) |
| Temperature, K | 150 |
| Space group | *C2*/*c* |
| No. of formula units per unit cell, *Z* | 8 |
| Radiation wavelength, Å | Mo *K*α, 0.71073 |
| Absorption coefficient, $\mu\cdot mm^{-1}$ | 0.087 |
| Collected refls. | 15927 |
| Independent Refls, $R_{int}$ | 2854, 0.037 |
| Observed refls. ($I > 2\sigma(I)$) | 2600 |
| Final $R_1$ values ($I > 2\sigma(I)$) | 0.077 |
| Final $wR(F^2)$ values (all data) | 0.170 |
| GOF | 1.00 |
| F000 | 1152 |

**Table S2.** Crystallographic data R values of Rietveld refinement and Pawley fit of the same pattern for [CBZ+MePRB] (1:1) form II cocrystal

| Compound reference | Pawley fit | Rietveld fit |
|---|---|---|
| CCDC number | | 2262554 |
| Chemical formula | | $C_{15}H_{12}N_2O\cdot C_8H_8O_3$ |
| *F*w | | 388.41 |
| Crystal system | | Monoclinic |
| *a,* Å | | 8.3140 (3) |
| *b*, Å | | 6.7122 (2) |
| *c*, Å | | 35.1490 (11) |
| *α*, ° | | 90.00 |
| *β*, ° | | 92.150 (11) |
| *γ*, ° | | 90.00 |
| Unit cell volume, Å$^3$ | | 1960.18 (13) |
| Temperature, K | | 298 |
| Space group | | $P2_1/c$ |
| No. of formula units per unit cell, *Z* | | 4 |
| Radiation wavelength, Å | | Synchrotron, λ = 0.75 Å |
| Absorption coefficient, $\mu\cdot mm^{-1}$ | | 0.10 |
| K1 | - | 1 |
| $R_{WP}$ | 0.61 | 1.01 |
| $R'_{WP}$ | 0.89 | 1.61 |
| $R_P$ | 0.42 | 0.69 |
| $R'_P$ | 0.64 | 1.14 |
| $R_{Bragg}$ | - | 0.63 |

**Table S3.** Calculated crystal lattice energies for form I and form II of [CBZ+MePRB] (1:1) derived from the periodic DFT computations with plane-wave basis sets. The units are kJ·mol$^{-1}$[a]

| Method | $E_{latt}$ (Form I) | $E_{latt}$ (Form II) | $\Delta E_{latt}$ |
|---|---|---|---|
| PBE-D3 | 243.8 | 245.0 | 1.2 |
| B86bPBE-XDM | 244.3 | 245.7 | 1.4 |

[a] $E_{latt} = \sum_{i=1}^{n} E_{mol,i} - \frac{E_{cry}}{Z}$, where $E_{mol}$ – total electronic energy of isolated molecule in relaxed conformations, $E_{cry}$ – total energy of the crystal, $Z$ - number of molecules in the unit cell.

**Table S4.** Experimental values of the CBZ ($[CBZ]_{eu}$) and MePRB ($[MePRB]_{eu}$) eutectic concentrations, calculated values of eutectic constant ($K_{eu}$) and the cocrystal solubility ($S_{CC}$) in a pH 6.5 buffer solution at 37 °C

| | $pH_{final}$ | $[CBZ]_{eu}$, M | $[MePRB]_{eu}$, M | $K_{eu}$[a] | $S_{CC}$,[b] M |
|---|---|---|---|---|---|
| [CBZ+MePRB] (1:1) Form II | 6.5 | $(1.12\pm0.03)\cdot10^{-3}$ | $(7.81\pm0.02)\cdot10^{-3}$ | 7.0±0.1 | $(2.96\pm0.04)\cdot10^{-3}$ |

[a] $K_{eu} = [MePRB]_{eu}/[CBZ]_{eu}$

[b] $S_{CC} = \sqrt{[CBZ]_{eu}[MePRB]_{eu}}$
